# Machine Learning-Based Multiclass Classification of Cognitive Stages Using Plasma Biomarkers, Clinical Assessments, and Genetic Features: A Repeated, Nested Cross-Validation Study in ADNI with External Evaluation in CNTN

**DOI:** 10.3390/diagnostics16121755

**Published:** 2026-06-06

**Authors:** Jiayuan Xu, Fumie Costen

**Affiliations:** Department of Electrical and Electronic Engineering, University of Manchester, Manchester M13 9PL, UK; jiayuan.xu@manchester.ac.uk

**Keywords:** Alzheimer’s disease, mild cognitive impairment, plasma biomarkers, machine learning, nested cross-validation, multiclass classification

## Abstract

**Background**: Plasma biomarkers are promoted as scalable tools for the staging of Alzheimer’s disease (AD), yet head-to-head comparisons against the clinical scales used to define diagnostic labels remain scarce. Reported gains from machine learning fusion of clinical and biomarker features may reflect label circularity rather than biological signals, and quantifying this circularity is a central aim of the present work. **Methods**: From the Alzheimer’s Disease Neuroimaging Initiative (ADNI), we assembled 655 participants (CN = 296, MCI = 168, and AD = 191) with concurrent plasma biomarkers (pT217, Aβ42/40, NfL, and GFAP), clinical scales (MMSE, CDR-SB, and FAQ), APOE genotype, and demographics. Three pre-specified feature sets (clinical-only, biomarker plus demographic–genetic, and full fusion) were compared across four classifiers (Logistic Regression, SVM, Random Forest, and XGBoost) using repeated, nested cross-validation (5-fold × 3 outer, 5-fold inner) with balanced class weighting. Because the external Center for Neurodegeneration and Translational Neuroscience (CNTN) cohort (n=130) measures pT181 rather than pT217 and lacks Aβ42/40, external evaluation used a separate reduced feature panel (NfL, GFAP, APOE, age, sex, and education), not the proposed pT217-inclusive panel. **Results**: Clinical scales alone reached a three-class AUC-OVR of 0.9539±0.0041, and fusion reached 0.9559±0.0046, an indistinguishable gain. Because MMSE, CDR-SB, and FAQ partly determine ADNI diagnostic labels, both estimates are circularity-inflated upper bounds and do not reflect independent classification power. Independent of this circularity, the internal plasma plus demographic–genetic model still achieved AUC-OVR =0.7455±0.0150, with pT217 as the dominant contributor. Pairwise discrimination was excellent for CN vs. AD (1.0000) and MCI vs. AD (0.9739) but markedly weaker for CN vs. MCI (0.9302 for fused and 0.6972 for plasma only). The separate reduced-feature model, which contains neither pT217 nor Aβ42/40, transferred to CNTN with AUC-OVR =0.702 (95% CI 0.635–0.764). **Conclusions**: Apparent fusion gains in ADNI are largely a consequence of label circularity. After removing the circular clinical features, the internal pT217-inclusive plasma model supports three-class CN/MCI/AD screening at AUC ≈0.74 and a reduced panel without pT217 transfers to an independent cohort at AUC ≈0.70. These values provide a realistic performance estimate for blood-based AD staging under the current feature set, diagnostic label structure, and cohort design, and richer feature sets or pathology-anchored labels may shift this estimate. MCI detection remains the principal bottleneck.

## 1. Introduction

Alzheimer’s disease (AD) is the most prevalent neurodegenerative disorder worldwide, affecting an estimated 55 million people globally and projected to exceed 150 million by 2050 [1,2]. The disease follows a protracted continuum, progressing from a preclinical phase of normal cognition (CN) through mild cognitive impairment (MCI) to AD dementia [3]. Early and accurate stratification along this continuum is critical for clinical trial enrichment, timely intervention, and the growing era of disease-modifying therapies [4].

Historically, AD staging has relied on costly or invasive procedures such as cerebrospinal fluid (CSF) analysis or amyloid positron emission tomography (PET) imaging [5]. The advent of ultrasensitive immunoassay platforms has enabled detection of AD-related proteins in peripheral blood at clinically actionable concentrations [6]. Among these, phosphorylated tau-217 (pT217), the amyloid-β 42/40 ratio (Aβ42/40), neurofilament light chain (NfL), and glial fibrillary acidic protein (GFAP) have each shown associations with AD pathology [7,8,9]. However, the extent to which these biomarkers can independently stratify patients across the full CN–MCI–AD spectrum, relative to established cognitive instruments, has not been fully characterized.

Machine learning (ML) models have been widely applied to AD classification tasks using imaging, genetic, and clinical data [10,11]. However, many prior studies have employed single train–test splits or simple cross-validation schemes that produce optimistic performance estimates, particularly in small-to-moderate cohorts [12]. Repeated, nested cross-validation (CV) mitigates this bias by strictly separating hyperparameter tuning from performance evaluation [13], yet few studies have applied it to plasma biomarker panels for three-class cognitive staging.

The APOE ε4 allele is the strongest known genetic risk factor for sporadic AD [14], and incorporating it alongside plasma biomarkers and demographic variables may improve classification beyond biomarkers alone. Whether such a composite biomarker model approaches clinical assessment-level performance has important implications for scalable, minimally invasive cognitive screening.

This comparison, however, is complicated by how diagnostic labels are defined. In ADNI, MMSE, CDR-SB, and FAQ are not independent of the diagnostic labels they are used to predict because the same instruments contribute to the assignment of CN, MCI, and AD status. A model built on these scales therefore partly re-derives the label definition, so clinical-only and biomarker-only performance measure different quantities and are not directly comparable. Making this circularity explicit and quantifying its contribution to the apparent fusion advantage are central objectives of this study.

The present study addresses three pre-specified objectives: (1) to evaluate whether plasma biomarkers augmented by demographic and genetic information can classify CN, MCI, and AD; (2) to compare this biomarker-based model against one relying exclusively on validated clinical scales; and (3) to determine whether the fusion of all feature types yields additional discriminative gain. These objectives are addressed using a repeated, nested CV protocol applied to the ADNI cohort, with external evaluation in the CNTN cohort and 95% confidence intervals reported for all performance estimates.

## 2. Materials and Methods

### 2.1. Data Sources

#### 2.1.1. ADNI Cohort

The primary analysis cohort was obtained from the Alzheimer’s Disease Neuroimaging Initiative (ADNI; https://adni.loni.usc.edu (accessed on 1 February 2026)) [15], a longitudinal multi-site study launched in 2003 to develop clinical, imaging, genetic, and biochemical biomarkers for the early detection and monitoring of AD. ADNI was approved by the institutional review boards of all participating institutions, and written informed consent was obtained from all participants or their authorized representatives.

ADNI phases included were ADNI 1, ADNI GO, ADNI 2, ADNI 3, and ADNI 4. Diagnosis records were downloaded in February 2026 (DXSUM_02Feb2026.csv). Plasma biomarker data were obtained from the University of Pennsylvania dataset using the Fujirebio Lumipulse (Fujirebio, Tokyo, Japan) and Quanterix Simoa (Quanterix, Billerica, MA, USA) platforms (UPENN_PLASMA_FUJIREBIO_QUANTERIX_02Feb2026.csv).

#### 2.1.2. CNTN Cohort

To assess the robustness and external generalizability of the ADNI-derived models, an independent dataset was obtained from the Center for Neurodegeneration and Translational Neuroscience (CNTN; https://nevadacntn.org/ (accessed on 6 January 2026)) data center [16], which is dedicated to studying neurodegenerative diseases in the aging population. The CNTN study protocols were approved by the Cleveland Clinic Institutional Review Board, and all participants provided written informed consent.

CNTN measures plasma pT181 rather than pT217 and does not include the plasma Aβ42/40 ratio, so external evaluation was performed on the intersection of variables available in both datasets (NfL, GFAP, APOE genotype, age, sex, and education). After applying inclusion criteria analogous to ADNI, the analytic external sample comprised n=130 participants (CN = 57, MCI = 56, and AD = 17).

### 2.2. Participants and Diagnostic Labels

Diagnosis codes follow the ADNI convention: 1 = CN, 2 = MCI, and 3 = AD. Eligible participants required concurrent availability of a valid diagnosis label, plasma biomarker concentrations (pT217, Aβ42/40, NfL, and GFAP), clinical assessments (MMSE, CDR-SB, and FAQ), APOE genotype, and demographic data (age, sex, and years of education). ADNI encodes missing or unavailable biomarker values as sentinel codes (−4.0 = not available; −5.0 = not done). All rows containing sentinel values in any plasma biomarker column were excluded prior to analysis. Plasma samples and diagnostic assessments were obtained at the same ADNI visit (matched by visit code VISCODE2). A total of 655 unique participants met all inclusion criteria: CN = 296, MCI = 168, and AD = 191.

### 2.3. Feature Variables

Three feature sets were pre-specified to enable systematic ablation:

*Clinical-only* (3 features): MMSE score, CDR-SB, and FAQ total score. These instruments constitute the backbone of cognitive staging in clinical practice and ADNI study criteria. Because the same instruments also inform ADNI diagnostic-label assignment, any model trained on this feature set is subject to the label circularity examined in Section 4.3.

*Biomarker plus demographic–genetic* (8 features): plasma pT217 (pg/mL), Aβ42/40 ratio, NfL (pg/mL), GFAP (pg/mL), APOE4 allele count (0, 1, or 2), age at examination (years), years of education, and sex (1 = male; 0 = female).

*Fusion* (11 features): the union of all variables from the two sets above.

### 2.4. Statistical Characterization of Participants

Between-group differences for continuous variables were assessed by one-way analysis of variance (ANOVA), with Tukey’s honestly significant difference (HSD) test applied post hoc for plasma biomarkers. Categorical variables (sex, APOE4 carrier status, and APOE4 homozygosity) were compared using Pearson’s chi-square test. All *p*-values are two-sided, and p<0.05 was considered statistically significant. To account for the multiple group comparisons reported in Table 1, Benjamini–Hochberg false-discovery-rate (FDR) correction was applied across these variables, and FDR-adjusted *p*-values are reported alongside nominal values. The model-level comparison between the fusion and clinical-only configurations rests on a single pre-specified test and is reported without further correction. The AUC-OVR, macro F1, balanced accuracy, and Brier estimates are descriptive quantities reported with 95% confidence intervals rather than hypothesis tests, so no multiplicity correction applies to them.

### 2.5. Machine Learning Classifiers

Four classifiers were evaluated: Logistic Regression (LR), Support Vector Machine (SVM), Random Forest (RF), and eXtreme Gradient Boosting (XGBoost). All models were implemented in Python version 3.14.4 (Python Software Foundation, https://www.python.org, accessed on 1 February 2026), using scikit-learn version 1.8.0 for LR, SVM, and RF, and xgboost version 3.2.0 for XGBoost. These models were selected to span a range from linear (LR and SVM) to non-linear ensemble architectures (RF and XGBoost), enabling comparison across model complexity levels [11].

### 2.6. Repeated, Nested Cross-Validation Protocol

Model evaluation used a repeated, nested CV protocol [12,13] that strictly separates hyperparameter tuning from performance evaluation to avoid optimistic bias.

*Outer loop:* RepeatedStratifiedKFold with 5 splits × 3 repetitions = 15 outer folds.

*Inner loop:* StratifiedKFold with 5 splits applied exclusively to outer training data for hyperparameter tuning via GridSearchCV. Each model was wrapped in a scikit-learn Pipeline consisting of a StandardScaler (*z*-score normalization), followed by the classifier, ensuring that scaling parameters were fit solely on training data within each fold and applied to the held-out test set, thereby preventing data leakage. For LR, SVM, and RF, class imbalance was handled via class_weight = ‘balanced’; for XGBoost, per-fold sample weights computed from sklearn.utils.class_weight.compute_sample_weight were passed as fit parameters. The two approaches are functionally equivalent.

*Hyperparameter search spaces:* LR searched C∈{0.1,1,10} and penalty $\in \{\ell_{1},\ell_{2}\}$ (SAGA solver). SVM searched C∈{0.5,1,5} and kernel ∈{linear, RBF}. RF searched estimators ∈{100,300}, max depth ∈{5,None}, and min samples split ∈{2,10}. XGBoost searched estimators ∈{100,300}, max depth ∈{3,5}, learning rate ∈{0.03,0.1}, and subsample ∈{0.8,1.0}. Inner-loop optimization used AUC-OVR for three-class tasks and standard AUC for binary tasks.

**Figure 1:**
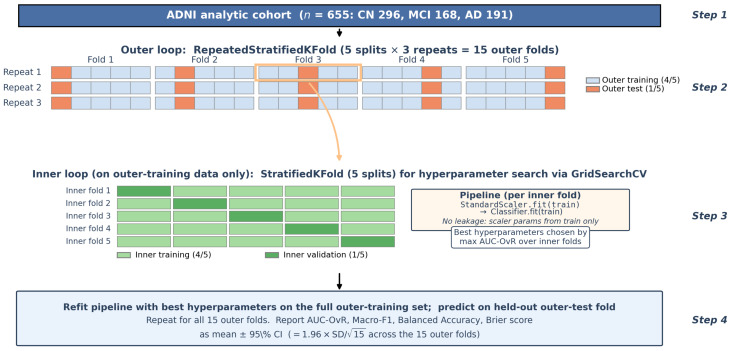
Schematic of the repeated, nested cross-validation protocol. **Step 1**: The full ADNI analytic cohort (n=655). **Step 2**: The outer loop uses RepeatedStratifiedKFold with 5 splits and 3 repeats, producing 15 distinct outer folds; in each fold, four-fifths of the data are retained for training (light blue), and one-fifth is held out as the unseen test set (orange). **Step 3**: Within each outer training set, a second StratifiedKFold (5 splits) is applied to perform GridSearchCV-based hyperparameter selection; for every inner fold, a pipeline fits the StandardScaler and the classifier exclusively on the inner training portion, preventing any information leakage from the inner validation split. **Step 4**: The pipeline is refit on the full outer training set with the best hyperparameters and evaluated on the held-out outer test fold; this is repeated for all 15 outer folds, and AUC-OVR, macro F1, balanced accuracy, and the Brier score are reported as mean ± 95% CI ($=1.96\times \mathrm{SD}/\sqrt{15}$).

The full repeated, nested protocol is summarized schematically in Figure 1. Three outer-loop repeats (yielding 15 outer folds in total) were used to attenuate the variance contributed by random fold partitioning: a single 5-fold split is, itself, a random draw, and pooling 15 fold-level estimates produces a more stable mean and a 95% confidence interval ($1.96\times \mathrm{SD}/\sqrt{15}$) that reflects both within-partition and between-partition variability [12].

### 2.7. Performance Metrics

On each outer test fold, three discrimination metrics were computed: (1) AUC-OVR (macro-averaged one-vs.-rest area under the ROC curve) for three-class tasks or standard binary AUC, (2) macro F1 score, and (3) balanced accuracy. Results are reported as mean ± 95% CI across 15 outer folds, where CI =$1.96\times \mathrm{SD}/\sqrt{15}$. As a calibration metric [17], (4) the multiclass Brier score was additionally computed for the three-class tasks (see Supplementary Note S1); binary Brier scores were computed for pairwise tasks. Brier-score results are reported in Supplementary Table S3 (multiclass) and Table S4 (pairwise).

### 2.8. Pairwise Binary Classification

Pairwise binary classifiers were trained on three tasks (CN vs. MCI, CN vs. AD, and MCI vs. AD) using the fusion feature set, applying the same repeated, nested CV protocol.

### 2.9. Plasma Biomarker Subset Analysis

To isolate the contribution of each plasma biomarker while controlling for demographic and genetic variables, all 15 non-empty subsets of {pT217, Aβ42/40, NfL, GFAP} were evaluated using a Random Forest classifier for the three-class (CN/MCI/AD) classification task. In each model, age, sex, education, and APOE4 allele count were included as fixed background features. Random Forest was selected for its strong performance across feature sets and its compatibility with SHAP-based interpretability.

### 2.10. Feature Interpretability via SHAP

SHAP (SHapley Additive exPlanations) values [18] were computed using the shap package (version 0.51.0) within the same repeated, nested cross-validation framework used for performance evaluation. For each of the 15 outer folds, a pipeline (StandardScaler + Random Forest with class_weight = ‘balanced’) was tuned by the same inner-loop 5-fold GridSearchCV used for performance evaluation (Section 2.6) and refit on the full outer training partition. TreeSHAP was then applied to the held-out outer test partition (out-of-fold SHAP) so that each fold’s SHAP values came from the same per-fold hyperparameter configuration used for the performance evaluation. For each fold, the global feature importance was obtained by averaging absolute SHAP values across both samples and classes, whereas per-class importance was obtained analogously by averaging across samples only (preserving the class axis). Fold-level values were then aggregated across the 15 outer folds and reported as mean ± 95% CI ($=1.96\times \mathrm{SD}/\sqrt{15}$). The same procedure was applied to both the biomarker plus demographic–genetic and fusion models. Beeswarm visualizations use out-of-fold SHAP averaged across the three test-fold appearances of each participant.

### 2.11. External Evaluation Procedure

To assess generalizability of the ADNI-derived models, we performed a reduced-feature external transfer to the CNTN cohort introduced in Section 2.1.2. Because CNTN does not measure pT217 or Aβ42/40, this procedure evaluates transfer of the residual NfL and GFAP signals and does not validate the proposed pT217-inclusive panel. We re-trained the ADNI model on the intersection of available features (NfL, GFAP, APOE ε4 allele count, age, sex, and education) and applied the resulting pipeline to CNTN without any fine-tuning (zero-shot transfer). For each classifier (LR, SVM, RF, and XGBoost), we performed 5-fold inner cross-validation on the full ADNI for hyperparameter selection, refit on all of ADNI and evaluated on CNTN. Multi-class AUC-OVR, macro F1, balanced accuracy, and the multi-class Brier score were computed. The 95% confidence intervals were obtained from 1000 stratified bootstrap resamples of the CNTN evaluation set.

## 3. Results

### 3.1. Participant Characteristics

Table 1 presents demographic, clinical, and biomarker characteristics for the 655 participants by diagnosis group.

CN participants were younger than MCI and AD participants (77.3 vs. 79.3 and 79.1 years; p=0.011) and had more years of education (16.7 vs. 16.0 and 16.1; p=0.008). All three clinical scales showed a monotonic gradient from CN through MCI to AD (all p<0.001): CDR-SB ranged from 0.1 (CN) to 7.4 (AD), and FAQ ranged from 0.4 (CN) to 18.9 (AD).

Among plasma biomarkers, pT217, NfL, and GFAP differed across groups (all p<0.001). The Aβ42/40 ratio showed a significant overall group difference (p=0.002), with a subtle decreasing trend from CN to AD consistent with amyloid pathology, although pairwise effect sizes were small.

APOE4 carrier rates were 36.1% (CN), 41.1% (MCI), and 64.4% (AD). APOE4 homozygosity rates were 3.0%, 8.9%, and 19.4% (both p<0.001). The proportions of female participants were 52.4% (CN), 44.6% (MCI), and 34.6% (AD; p<0.001).

### 3.2. Three-Class Classification Performance

Table 2 presents the AUC-OVR, macro F1, and balanced accuracy from the repeated, nested CV experiment across all three feature sets and four classifiers.

All four classifiers using the clinical-only feature set achieved an AUC-OVR between 0.9449 and 0.9539. Logistic Regression performed best (0.9539±0.0041, macro F1 =0.8510±0.0114). The biomarker plus demographic–genetic set achieved AUC-OVR values of 0.7356–0.7455 and macro-F1 values of 0.55–0.57 across all classifiers. Adding clinical scales to form the fusion set restored AUC-OVR to the clinical-only range, and XGBoost reached the highest value (0.9559±0.0046). Neither the Wilcoxon signed-rank test (W=42, p=0.33) nor the paired *t*-test ($t_{14}=1.31$, p=0.21) detected a difference between the fusion and clinical-only models.

### 3.3. Pairwise Binary Classification Results

Table 3 presents pairwise binary classification results using the fusion feature set across all three diagnostic contrasts.

CN vs. AD discrimination achieved AUC ≥0.9997 across all classifiers. However, this near-perfect result is largely attributable to the extreme separation provided by CDR-SB and FAQ between CN and AD groups (see Section 4.3 for discussion of definitional circularity). MCI vs. AD AUC ranged from 0.9682 (XGBoost) to 0.9739 (Logistic Regression). CN vs. MCI was the most difficult task (Logistic Regression AUC =0.9302±0.0126). For comparison, using plasma biomarkers alone (without clinical scales), pairwise AUCs were 0.6972 (CN vs. MCI), 0.9153 (CN vs. AD), and 0.7588 (MCI vs. AD), indicating that the high pairwise performance reported in Table 3 is driven by the clinical-scale components of the fusion set. Aggregated confusion matrices (Supplementary Table S5) confirm that MCI is the primary source of misclassification: the Random Forest fusion model achieved an MCI recall of 73.0%, compared with 88.3% for CN and 91.4% for AD. The per-class breakdown of MCI errors across feature sets is summarized in Table 4.

### 3.4. Plasma Biomarker Subset Analysis Results

Table 5 presents selected results from the systematic enumeration of all 15 plasma biomarker subsets evaluated for the three-class (CN/MCI/AD) classification task using Random Forest with fixed background features.

**Figure 2:**
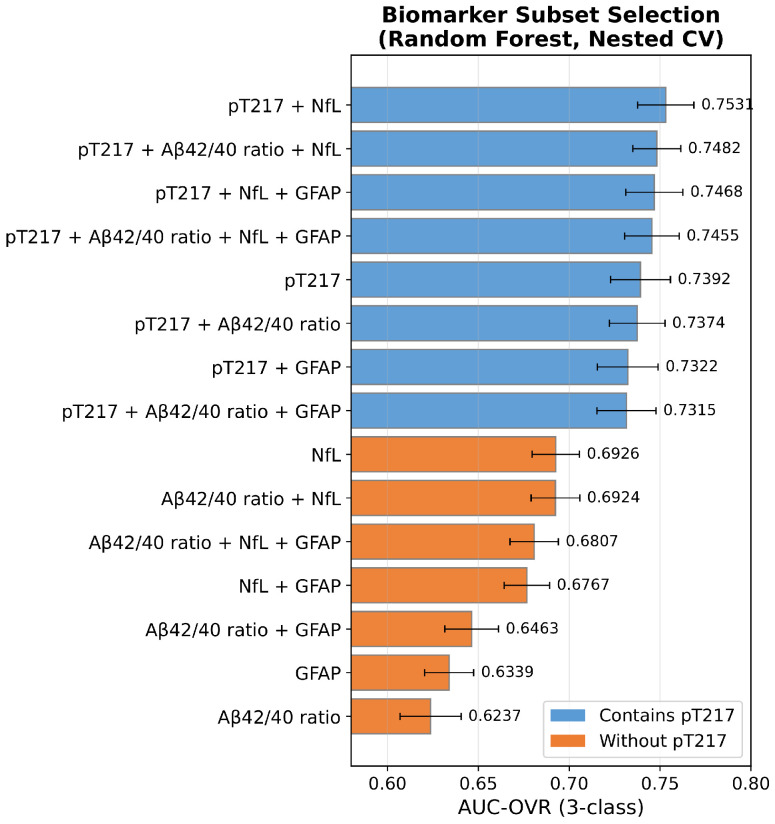
Biomarker subset selection results. Mean three-class AUC-OVR (repeated, nested cross-validation with 15 outer folds) for all 15 non-empty subsets of {pT217, Aβ42/40, NfL, and GFAP} evaluated by Random Forest with fixed background features. The consistent dominance of pT217-containing panels and the limited contribution of Aβ42/40 are apparent.

Three key findings emerged from the subset analysis. First, pT217 alone achieved the highest single-marker three-class AUC-OVR of 0.7392±0.0165. Second, the two-marker combination of pT217 + NfL was the best-performing subset (AUC-OVR =0.7531±0.0155), followed by pT217 + NfL + GFAP (AUC-OVR =0.7468±0.0157) and the full four-biomarker panel (AUC-OVR =0.7455±0.0150). Third, adding Aβ42/40 to any subset did not improve and, in some cases, reduced AUC-OVR by 0.002–0.005, consistent with the small pairwise effect sizes of Aβ42/40 (one-way ANOVA p=0.002; Tukey HSD: AD vs. CN p=0.002, but CN vs. MCI p=0.18 and MCI vs. AD p=0.33). Figure 2 displays the results for all 15 subsets: pT217-containing panels consistently outperformed those without pT217, while Aβ42/40-only subsets produced the lowest AUC-OVR values.

### 3.5. Feature Interpretability via SHAP Results

**Figure 3:**
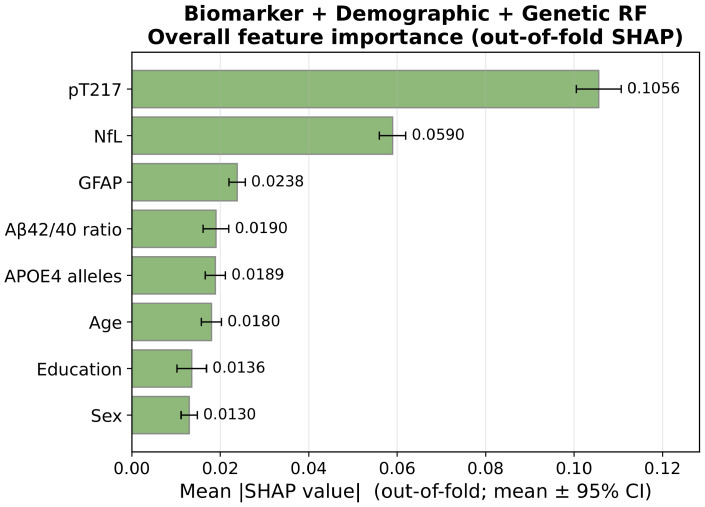
Overall feature importance from SHAP analysis (mean absolute SHAP values averaged across CN, MCI, and AD classes) for the biomarker plus demographic–genetic Random Forest model aggregated across the 15 outer cross-validation folds (mean ± 95% CI; error bars). pT217 dominates global importance by a wide margin, followed by NfL, GFAP, Aβ42/40, and age.

**Figure 4:**
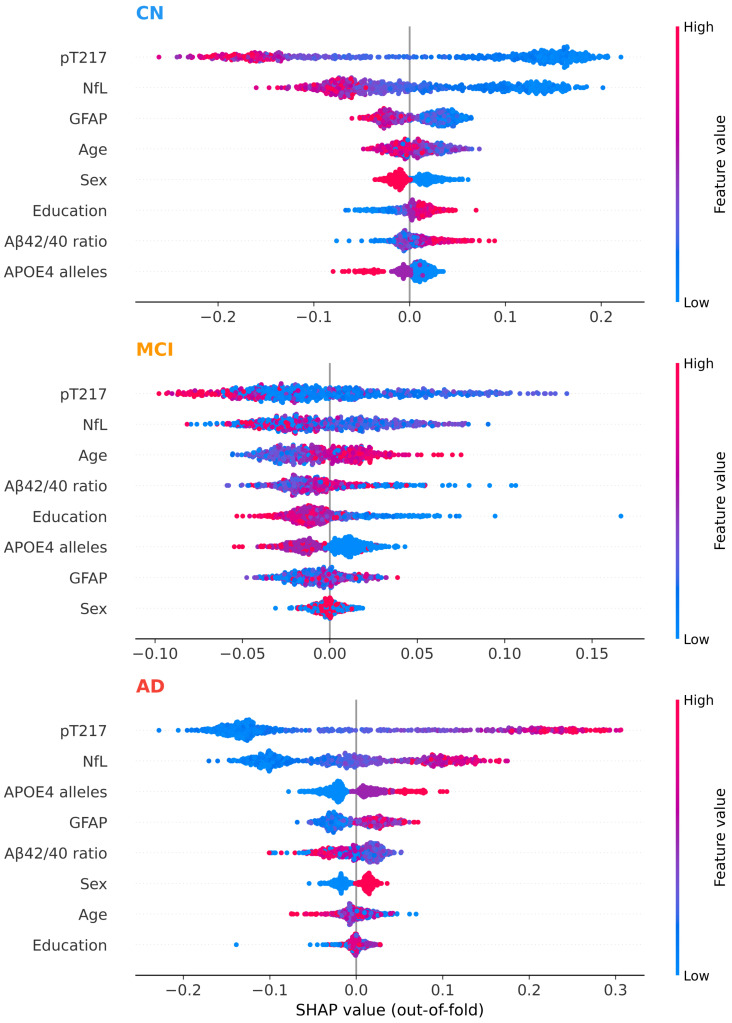
Class-specific SHAP beeswarm plots for CN (**top**), MCI (**middle**), and AD (**bottom**). Each point represents a participant’s out-of-fold SHAP value averaged across the three outer test folds in which the participant appeared (15 outer folds total). pT217 is the dominant feature across all three classes: high pT217 values increase AD prediction probability and decrease CN probability.

**Figure 5:**
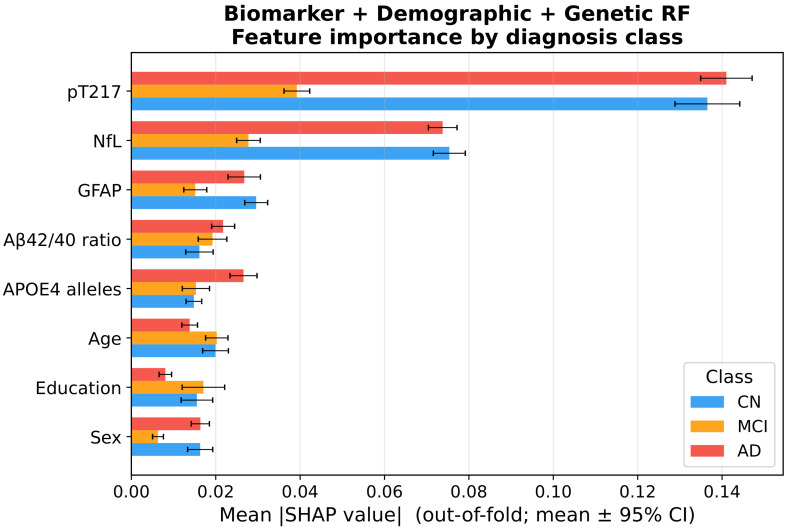
Mean absolute SHAP values by feature and diagnostic class (CN, MCI, and AD) aggregated across the 15 outer cross-validation folds (mean ± 95% CI; error bars). pT217 and NfL show the highest class-differentiated importance.

To quantify each feature’s contribution to individual predictions, SHAP values were aggregated across the 15 outer cross-validation folds (out-of-fold SHAP, mean ± 95% CI; see Methods Section 2.10). Each SHAP value quantifies how much a feature pushes a prediction toward or away from a particular class. Larger absolute values indicate stronger influence. Figure 3 shows that pT217 dominates global feature importance, with a mean absolute SHAP of 0.106±0.005, which is 1.8 times larger than NfL (0.059±0.003). GFAP (0.024±0.002), Aβ42/40 (0.019±0.003), APOE4 (0.019±0.002), age (0.018±0.002), education (0.014±0.003), and sex (0.013±0.002) each contributed smaller effects. Figure 4 displays class-specific beeswarm plots: high pT217 values (red dots) shift leftward in the CN panel (decreasing CN probability) and rightward in the AD panel (increasing AD probability), with an attenuated pattern for MCI. Figure 5 decomposes mean absolute SHAP values by diagnostic class, showing that pT217 importance is highest for CN and AD but reduced for MCI, consistent with the difficulty of MCI detection.

Demographic variables (age, education, and sex) and APOE4 allele count each contributed mean absolute SHAP values ≤0.019, comparable to Aβ42/40 and notably smaller than pT217, NfL, and GFAP. Sex showed a larger SHAP contribution for CN than for MCI or AD. This class-specific effect requires replication in larger, sex-stratified cohorts.

To quantify the influence of definitional circularity on the fusion model, we applied the same SHAP analysis to the Random Forest fusion model (11 features) aggregated across the 15 outer cross-validation folds. CDR-SB alone accounted for 41.7% of the total mean absolute SHAP value, followed by FAQ (23.0%) and MMSE (13.6%), for a combined 78.3% of the total feature importance from the three clinical scales. Among plasma biomarkers, pT217 was the largest contributor (9.1%), followed by NfL (4.6%), GFAP (2.2%), and Aβ42/40 (1.3%). Demographic and genetic variables (age, education, APOE4, and sex) collectively accounted for 4.6%. These results demonstrate that the fusion model’s performance is overwhelmingly driven by the clinical scales that participate in diagnostic label assignment, reinforcing the interpretation that a clinical-only AUC near 0.95 reflects definitional circularity rather than independent classification power (Supplementary Figure S1).

### 3.6. Reduced-Feature External Transfer to the CNTN Cohort

The reduced ADNI model transferred to CNTN with discrimination retained near the level achieved on ADNI itself with the same feature panel (Table 6). The best transferring model was SVM, which achieved AUC-OVR =0.702 (95% CI 0.635–0.764) on CNTN versus 0.687 in inner ADNI CV, indicating that discrimination was preserved when the ADNI-trained reduced model was applied to CNTN, with no significant cross-cohort transfer loss under this matched feature panel. Per-class behavior mirrored the patterns reported for ADNI in Section 3.3: CN was recovered with high recall (SVM: 77%, 44/57), while MCI, again, underperformed (27%, 15/56; Table 7), reproducing the same heterogeneity-driven pattern in an independent cohort. Because the CNTN AD subgroup is small (n=17), per-class estimates for AD carry wide uncertainty, and the bootstrap confidence intervals in Table 6 are the primary indicator of this imprecision. The CNTN results are therefore best read as preliminary evidence that the discriminative pattern transfers, not as confirmation of generalizability.

## 4. Discussion

### 4.1. Comparison with Prior Studies

To situate our findings within the broader literature, Table 8 summarizes representative plasma biomarker and ADNI-based machine learning studies. Because these studies differ from ours in endpoint definition, input modality, and validation strategy, the comparison is intended to be contextual rather than a direct benchmark.

Three observations emerge from Table 8. First, the high AUCs of 0.92–0.98 reported by Ashton et al. [7] and Barthélemy et al. [19] were obtained against pathology-defined endpoints (amyloid-PET or CSF positivity) rather than clinical diagnostic categories. These studies establish that plasma pT217 tracks underlying AD pathology with high fidelity, and our SHAP results (in which pT217 dominates plasma biomarker importance) are consistent with this evidence. However, predicting pathology positivity is a fundamentally different and easier task than discriminating clinically heterogeneous CN, MCI, and AD groups, so the two sets of AUC values are not interchangeable measures of model utility.

Second, prior ADNI machine learning studies differ from the present work in both input modality and task formulation. Bron et al. [21] used structural MRI for binary CN vs. AD classification, Zhao et al. [22] used tau-PET radiomics for pairwise CN vs. MCI and MCI vs. AD tasks, and Cai et al. [20] addressed longitudinal conversion within fixed clinical strata. The present study uses peripheral plasma biomarkers, together with demographics and APOE4, to classify all three clinical categories simultaneously, defining a distinct endpoint–modality combination relative to these prior reports.

Third, the evaluation protocol also differs. Most prior studies relied on a single train–test split or simple *k*-fold cross-validation, both of which are known to yield optimistic and high-variance estimates in small or class-imbalanced cohorts [12,13]. The repeated, nested cross-validation protocol used here, including 15 outer folds with three inner-fold repetitions, decouples hyperparameter tuning from performance estimation and yields more reliable estimates.

### 4.2. SHAP Interpretability and Clinical Meaning

The SHAP analysis, aggregated across the 15 outer cross-validation folds (out-of-fold SHAP), confirms the biomarker subset ranking. pT217 achieved a mean absolute SHAP value of 0.106±0.005, which is 1.8 times larger than NfL (0.059±0.003). High pT217 values increased AD prediction probability and decreased CN probability, with an attenuated effect for MCI. This directional pattern is consistent with the known biology, as tau phosphorylation at threonine-217 correlates with amyloid plaque density and neurofibrillary tangle burden [7].

### 4.3. Clinical Scales Dominate Three-Class Performance

A key interpretive constraint is the definitional circularity between clinical-scale scores and ADNI diagnostic labels: MMSE, CDR-SB, and FAQ are, themselves, the principal instruments used to define diagnostic group membership [3]. Clinical diagnoses in ADNI incorporate CDR-global cutoffs directly (e.g., CDR-global =0 for CN, CDR-global ≥0.5 for MCI, and CDR-global ≥1.0 with cognitive impairment for AD), and MMSE and FAQ scores further inform the diagnostic algorithm. As a result, a substantial portion of the clinical-scale AUC of about 0.95 reflects this definitional overlap rather than independent predictive signals. This circularity means that clinical-only and biomarker-only AUC values reflect fundamentally different quantities and should not be directly compared. The biomarker AUC of 0.75 is free of this confound and therefore provides a more interpretable measure of genuine biological classification power.

Adding individual clinical scales to the biomarker set (Supplementary Table S2) showed that CDR-SB alone raised the AUC from 0.7455 to 0.9502, FAQ alone raised it to 0.8904, and MMSE alone raised it to 0.8421. The fusion model (AUC =0.9559, XGBoost) did not differ from clinical scales alone (AUC =0.9539, Logistic Regression; Wilcoxon W=42, p=0.33; paired *t*-test $t_{14}=1.31$, p=0.21).

Analysis of the SHAP fusion model provides direct evidence of this circularity. CDR-SB alone contributed 41.7% of total feature importance, and the three clinical scales, combined, accounted for 78.3%, substantially exceeding all plasma biomarkers combined (17.1%) and demographic/genetic variables (4.6%). This confirms that the fusion model’s performance is primarily attributable to the features used to define the diagnostic labels. Importantly, biomarker-only models provide an estimate of biological classification power that is free from this definitional confound, offering a more interpretable benchmark of genuine discriminative ability (AUC-OVR =0.75).

### 4.4. The Discriminative Value of Plasma Biomarkers

Within the modest three-class AUC of 0.74 achieved by the full plasma panel, subset analysis (Table 5) showed that pT217 alone already attained three-class AUC-OVR =0.74, and the pT217 + NfL combination reached 0.75, indicating that pT217 carries virtually the entire discriminative signal of the plasma panel.

The CN vs. MCI task remained the most challenging for plasma biomarkers (see Supplementary Note S2) (pT217 + NfL three-class AUC =0.75). The pattern of MCI misclassification is informative: aggregating the Random Forest biomarker-only confusion matrices across all 15 outer folds (168 MCI participants × 3 repeats =504 MCI test instances), 244 (48.4%) were classified as CN, 160 (31.7%) as AD, and only 100 (19.8%) correctly identified as MCI. Errors fell predominantly toward the CN class, with 60.4% of errors being false negatives, consistent with the clinical overlap between early MCI and cognitively normal aging.

The Aβ42/40 ratio showed a significant but small between-group difference (p=0.002) and provided limited additional classification value beyond pT217-containing panels [5,6].

### 4.5. APOE ε4 and Genetic Risk Architecture

APOE4 carrier prevalence ranged from 36.1% (CN) to 64.4% (AD), consistent with prior genetic epidemiology of AD [14]. APOE4 homozygosity was present in 19.4% of AD participants, versus 3.0% in CN.

### 4.6. Methodological Strength and Rigor

This study applies repeated, nested CV to a plasma biomarker classification problem and reports 95% confidence intervals across all metrics. The CNTN external evaluation (Section 3.6) provides additional evidence that the discriminative pattern is not specific to ADNI and partially generalizes to an independent cohort with overlapping features.

### 4.7. Biomarker Choice and External Transferability

The external cohort measures pT181 rather than pT217, and the two phosphorylated tau species differ in both analytical and biological behavior. Phosphorylation at threonine-217 shows a larger fold change between amyloid-positive and amyloid-negative individuals and tracks early amyloid accumulation more closely than pT181, which yields a wider dynamic range and stronger separation of diagnostic groups [6,7,19]. Because the internal model’s discriminative signal is concentrated in pT217 (Section 3.4 and Section 3.5), a transfer model that omits pT217 and relies on NfL and GFAP is expected to capture only part of that signal. The CNTN result (AUC-OVR =0.702) should therefore be read as a conservative estimate of what a pT217-inclusive panel might achieve in the same cohort, and the modest internal-to-external change does not imply that the full panel would transfer without loss. Direct external evaluation of a pT217-inclusive panel is the most important outstanding validation step.

### 4.8. Limitations

Several limitations warrant consideration. The analytic sample (n=655) excluded 92 participants with missing biomarker data (ADNI sentinel values), who were, on average, younger, more likely female, and less likely to carry APOE4, suggesting potential selection bias toward a more clinically impaired sample. A formal comparison of included and excluded participants is provided in Supplementary Table S6; all standardized mean differences were small (|SMD|≤0.32). A missing-data sensitivity analysis (Supplementary Note S3, Table S7), in which the excluded participants’ biomarkers were median-imputed and the model was re-evaluated, changed the three-class AUC-OVR by, at most, 0.009 across classifiers, confirming that the main result is robust to this exclusion. ADNI enrolls a predominantly non-Hispanic White, highly educated volunteer population from specialty memory clinics, which limits generalizability to community-based or ethnically diverse populations. Clinical-stage distributions in ADNI may not reflect the prevalence ratios encountered in primary care screening settings.

By design, this study focused exclusively on plasma-based and clinical measures, excluding neuroimaging data. While incorporating structural MRI or amyloid PET could potentially improve classification, the present pipeline prioritizes accessibility and cost-effectiveness for primary care screening settings. The CN vs. AD results (AUC ≥0.9997) using the fusion set are largely driven by clinical scales. However, the biomarker plus demographic–genetic set alone achieved CN vs. AD AUC =0.9153±0.0134 (Supplementary Table S1), confirming that CN and AD are also well separated on biological grounds. The group comparisons presented in Table 1 were corrected for multiple testing using the Benjamini–Hochberg procedure, and all remained significant. The remaining performance metrics are reported as descriptive estimates with 95% confidence intervals rather than as hypothesis tests, so no further multiplicity correction was applied. Multiclass and binary Brier scores are reported in Supplementary Tables S3 and S4. Calibration analysis (Supplementary Figure S2) showed good agreement across all three classes for the clinical-only and fusion models, whereas the biomarker-only model exhibited moderate miscalibration for MCI, consistent with the limited separability of this intermediate class using plasma markers alone. The present analysis is cross-sectional and therefore characterizes diagnostic group separation at a single time point rather than progression. The central findings of this work are properties of the feature space and are therefore independent of study design: the quantification of the definitional circularity that inflates the clinical-scale-driven AUC, the estimation of a plasma-alone three-class performance level near 0.75 under the current feature set and label definition, and the demonstration that pT217 carries virtually all of the plasma-discriminative signal. Extending the framework to predict MCI-to-AD conversion in ADNI’s longitudinal follow-up is a natural next step. A further intrinsic limitation is endpoint dependence: the diagnostic labels are defined, in part, by the same clinical instruments that the fusion model uses as inputs, so any performance estimate driven by the clinical scales is conditioned on the label-definition procedure rather than on an independent ground truth. The plasma-only estimate avoids this confound but is, in turn, bounded by the diagnostic labels available in ADNI, which are, themselves, clinical rather than neuropathological endpoints.

CNTN measures pT181 rather than pT217 and does not include plasma Aβ42/40. Accordingly, the external evaluation in Section 3.6 was based on the reduced-feature intersection of the two cohorts (NfL, GFAP, APOE, age, sex, and education) and therefore does not validate the proposed pT217-inclusive panel. The external sample is also small, particularly for AD (n=17), so the CNTN estimates carry wide confidence intervals and should be treated as preliminary. Strict external replication of the full pT217-inclusive panel in a larger, demographically broader cohort remains outstanding and is the single most important priority for future work.

## 5. Conclusions

Clinical assessment scales (MMSE, CDR-SB, and FAQ) achieved three-class AUC-OVR values around 0.95 in the ADNI cohort, compared with 0.75 for plasma biomarkers augmented by demographic and genetic variables. The fusion of all feature types produced the highest three-class AUC (XGBoost: 0.9559±0.0046), matching the performance of clinical scales alone (Wilcoxon p=0.33). An important methodological consideration is that MMSE, CDR-SB, and FAQ overlap with the instruments used to assign ADNI diagnostic labels, so the clinical-only AUC partly reflects this definitional relationship. In contrast, plasma biomarker performance (AUC-OVR =0.75) is free of this confound and therefore provides a more meaningful measure of biological classification accuracy.

Among plasma biomarkers, pT217 achieved the highest single-marker three-class AUC-OVR (0.74), and pT217 + NfL was the best two-marker combination (AUC-OVR =0.75). External evaluation in the CNTN cohort (n=130) used a reduced panel containing neither pT217 nor Aβ42/40 and showed that the residual NfL and GFAP signals transfer to an independent cohort (AUC-OVR =0.702 with the linear SVM), with the same per-class pattern observed in ADNI: high CN recall and low MCI recall. Given the small size of the external sample, this transfer result is preliminary, and the reported figures are best read as a realistic performance estimate under the current feature set, label structure, and cohort design, and richer feature sets or pathology-anchored labels may shift this estimate. MCI detection through plasma biomarkers alone remains limited and is the principal target for future work.

Future directions should include: (1) validation in ethnically diverse, population-based cohorts; (2) longitudinal extension to ADNI follow-up data for MCI-to-AD conversion prediction; and (3) inclusion of newer generations of biomarkers (e.g., pT231, and synaptic proteins).

## Figures and Tables

**Table 1:** Demographic, clinical, and biomarker characteristics of study participants stratified by diagnosis (one sample per participant).

Variable	CN (n=296)	MCI (n=168)	AD (n=191)	*p*-Value	FDR-Adj. *p*
*Demographic and clinical*					
Age (years)	77.3±7.8	79.3±7.5	79.1±8.0	0.011	0.011
Education (years)	16.7±2.4	16.0±2.7	16.1±2.6	0.008	0.009
MMSE score	28.8±1.5	27.0±2.7	19.1±6.3	<0.001	<0.001
CDR-SB	0.1±0.3	1.5±1.2	7.4±3.5	<0.001	<0.001
FAQ total	0.4±1.1	3.9±5.0	18.9±7.4	<0.001	<0.001
*Plasma biomarkers*					
pT217 (pg/mL)	0.2±0.2	0.4±0.3	0.8±0.6	<0.001	<0.001
Aβ42/40 ratio	0.085±0.013	0.083±0.011	0.081±0.012	0.002	0.003
NfL (pg/mL)	20.7±13.6	26.4±14.5	35.7±24.5	<0.001	<0.001
GFAP (pg/mL)	184.2±102.0	231.0±140.4	287.8±175.1	<0.001	<0.001
*Genetic and sex*					
Female sex, *n* (%)	155 (52.4%)	75 (44.6%)	66 (34.6%)	<0.001	<0.001
APOE4 carrier, *n* (%)	107 (36.1%)	69 (41.1%)	123 (64.4%)	<0.001	<0.001
APOE4 homozygous, *n* (%)	9 (3.0%)	15 (8.9%)	37 (19.4%)	<0.001	<0.001

Continuous variables: mean ± SD; one-way ANOVA *p*-value. Categorical variables: *n* (%); Pearson chi-square *p*-value. FDR-adjusted *p*-values use the Benjamini–Hochberg procedure across all variables in this table; every comparison remained statistically significant after correction ($p_{\mathrm{FDR}}<0.05$). Abbreviations: CN, cognitively normal; MCI, mild cognitive impairment; AD, Alzheimer’s disease; MMSE, Mini-Mental State Examination; CDR-SB, Clinical Dementia Rating Sum of Boxes; FAQ, Functional Activities Questionnaire; pT217, plasma phosphorylated tau-217; Aβ42/40, amyloid-β 42/40 ratio; NfL, neurofilament light chain; GFAP, glial fibrillary acidic protein.

**Table 2:** Three-class (CN/MCI/AD) repeated, nested cross-validation performance by feature set and classifier (n=655; 15 outer folds).

Feature Set	Classifier	AUC-OVR (95% CI)	Macro F1 (95% CI)	Bal. Acc. (95% CI)
*Clinical-only (3 features: MMSE, CDR-SB, and FAQ)*				
	Logistic Regression	0.9539±0.0041	0.8510±0.0114	0.8490±0.0121
	Random Forest	0.9490±0.0044	0.8574±0.0119	0.8609±0.0119
	SVM	0.9498±0.0056	0.8430±0.0105	0.8392±0.0104
	XGBoost	0.9449±0.0063	0.8589±0.0125	0.8621±0.0127
*Biomarker + Demographic + Genetic (8 features)*				
	Logistic Regression	0.7384±0.0200	0.5560±0.0263	0.5608±0.0266
	Random Forest	0.7455±0.0150	0.5608±0.0145	0.5752±0.0161
	SVM	0.7356±0.0197	0.5548±0.0238	0.5633±0.0233
	XGBoost	0.7388±0.0157	0.5654±0.0177	0.5749±0.0196
*Fusion (11 features: all variables combined)*				
	XGBoost	0.9559±0.0046 ^†^	0.8519±0.0101	0.8555±0.0101
	Random Forest	0.9538±0.0056	0.8467±0.0125	0.8497±0.0130
	Logistic Regression	0.9494±0.0036	0.8399±0.0088	0.8367±0.0086
	SVM	0.9495±0.0049	0.8345±0.0124	0.8322±0.0126

Results are mean ± 95% CI (=1.96 $\times \mathrm{SD}/\sqrt{15}$) across 15 outer folds. Bold indicates the numerically highest AUC. ^†^ Fusion XGBoost vs. Clinical-only Logistic Regression: Wilcoxon signed-rank test (W=42, p=0.33); paired *t*-test ($t_{14}=1.31$, p=0.21). The difference is not statistically significant.

**Table 3:** Pairwise binary classification performance using the fusion feature set (11 features; repeated, nested cross-validation with 15 outer folds). Note that the fusion set includes clinical assessment scales; results for the biomarker plus demographic–genetic set alone are provided in Supplementary Table S1.

Task	Classifier	AUC (95% CI)	F1 (95% CI)	Bal. Acc. (95% CI)
*CN vs. AD*				
	XGBoost	1.0000±0.0000	0.9948±0.0034	0.9957±0.0029
	Random Forest	1.0000±0.0001	0.9939±0.0034	0.9951±0.0028
	SVM	0.9997±0.0003	0.9835±0.0077	0.9863±0.0063
	Logistic Regression	0.9998±0.0003	0.9830±0.0075	0.9835±0.0072
*CN vs. MCI*				
	Logistic Regression	0.9302±0.0126	0.8162±0.0293	0.8527±0.0229
	Random Forest	0.9291±0.0106	0.8280±0.0180	0.8680±0.0154
	XGBoost	0.9282±0.0119	0.8280±0.0200	0.8680±0.0169
	SVM	0.9271±0.0154	0.8289±0.0234	0.8667±0.0193
*MCI vs. AD*				
	Random Forest	0.9719±0.0075	0.9113±0.0125	0.9045±0.0134
	Logistic Regression	0.9739±0.0069	0.9018±0.0156	0.8986±0.0158
	SVM	0.9722±0.0063	0.8981±0.0164	0.8921±0.0177
	XGBoost	0.9682±0.0070	0.9034±0.0166	0.8972±0.0163

Results are mean ± 95% CI across 15 outer folds. Bold indicates the best AUC per task.

**Table 4:** Per-class disposition of MCI test instances by feature set, aggregated across all 15 outer folds (168 MCI participants × 3 repeats =504 MCI test instances). Values are counts with row percentages; the diagonal column (MCI → MCI) is the correctly classified MCI count.

Feature Set (Model)	MCI → CN	MCI → MCI	MCI → AD	MCI Recall
Biomarker + Demo + Genetic (RF)	244 (48.4%)	100 (19.8%)	160 (31.7%)	19.8%
Clinical-only (LR)	102 (20.2%)	359 (71.2%)	43 (8.5%)	71.2%
Fusion (RF)	81 (16.1%)	368 (73.0%)	55 (10.9%)	73.0%

Counts aggregated across 15 outer folds (5-fold × 3 repeats); each MCI participant appears in 3 test folds. For the plasma-only model, MCI errors fall predominantly toward CN (false negatives), consistent with clinical overlap between early MCI and normal aging. Adding clinical scales (fusion) raises MCI recall to 73.0% but does not resolve the residual confusion with CN and AD. Source counts are given in Supplementary Table S5.

**Table 5:** Selected plasma biomarker subsets: three-class (CN/MCI/AD) AUC-OVR with fixed background features (Random Forest; 15 outer folds).

Biomarker Subset	CN/MCI/AD AUC-OVR
pT217 only	0.7392±0.0165
Aβ42/40 only	0.6237±0.0168
NfL only	0.6926±0.0130
GFAP only	0.6339±0.0135
**pT217 + NfL**	0.7531±0.0155
pT217 + NfL + GFAP	0.7468±0.0157
All four biomarkers	0.7455±0.0150

Results are mean ± 95% CI (15 outer folds). Bold indicates the best-performing subset. Background features fixed in all models: age, sex, years of education, and APOE4 allele count.

**Table 6:** Zero-shot transfer of the ADNI-trained reduced biomarker model (NfL + GFAP + APOE + age + sex + education) to CNTN (n=130: CN 57/MCI 56/AD 17). The 95% CIs from 1000-iter bootstrap.

Model	ADNI Inner CV AUC	CNTN AUC-OVR (95% CI)	Macro F1 (95% CI)	Bal. Acc. (95% CI)	Brier (95% CI)
Logistic Regression	0.692	0.675 (0.603–0.741)	0.407 (0.334–0.484)	0.456 (0.360–0.552)	0.203 (0.188–0.217)
SVM (linear)	0.687	**0.702 (0.635–0.764)**	0.460 (0.382–0.539)	0.503 (0.411–0.594)	0.197 (0.179–0.213)
Random Forest	0.680	0.645 (0.573–0.715)	0.400 (0.311–0.483)	0.470 (0.370–0.566)	0.204 (0.188–0.222)
XGBoost	0.666	0.623 (0.548–0.691)	0.409 (0.323–0.485)	0.464 (0.361–0.558)	0.213 (0.195–0.231)

Bold indicates the highest AUC-OVR.

**Table 7:** CNTN confusion matrix for SVM (best transferring model).

True\Predicted	CN	MCI	AD
CN	44	9	4
MCI	18	15	23
AD	2	7	8

**Table 8:** Contextual comparison of selected plasma biomarker and ADNI classification studies grouped by endpoint and modality.

Study	Cohort	Endpoint/Task	Modality	Validation	Performance
*Plasma biomarker studies (pathology endpoints)*					
Ashton et al. 2024 [7]	Multi-cohort (n=786)	Aβ-PET/tau-PET pathology positivity	Plasma pT217 (ALZpath)	Biomarker cutoff	0.92–0.97 (AUC)
Barthélemy et al. 2024 [19]	2 cohorts (n=1759)	Aβ-PET/tau-PET pathology positivity	Plasma %p-tau217 (Mass spec)	Predefined cutoff	0.95–0.98 (AUC)
*Present study, ADNI (n=655), repeated nested CV (15 outer folds)*					
Present study	ADNI (n=655)	CN/MCI/AD	Plasma + demographics + APOE4	Repeated nested CV (RF)	0.7455 (AUC)
Present study	ADNI (n=655)	CN/MCI/AD	Clinical scales	Repeated nested CV (LR)	0.9539 (AUC)
Present study	ADNI (n=655)	CN vs. AD	pT217 + NfL + background	Repeated nested CV (RF)	0.9238 (AUC)
*ADNI machine learning classification studies*					
Cai et al. 2023 [20]	ADNI (n=589)	4-year conversion (CU/MCI strata)	Plasma p-tau + NfL + clinical + APOE4	Cross-validation	0.65/0.80 (AUC)
Bron et al. 2021 [21]	ADNI (n=854)	CN vs. AD	Structural MRI	Nested CV (SVM)	0.940 (AUC)
Zhao et al. 2022 [22]	ADNI (n=525)	CN vs. MCI/MCI vs. AD	Tau-PET radiomics	DLR + SVM	0.908/0.884 (Accuracy)

Studies are grouped by endpoint and modality. Background features = age, sex, education, and APOE4 allele count. Abbreviations: LR, Logistic Regression; RF, Random Forest; SVM, Support Vector Machine; DLR, Deep Learning Radiomics; CV, cross-validation.

## Data Availability

ADNI data are publicly available to qualified researchers at https://adni.loni.usc.edu (accessed on 1 February 2026) upon completion of a data use agreement. CNTN data are available from the Center for Neurodegeneration and Translational Neuroscience (https://nevadacntn.org/ (accessed on 6 January 2026)). The Python analysis code is available from the corresponding author upon reasonable request.

## Supplementary Materials

The following supporting information can be downloaded at: https://www.mdpi.com/article/10.3390/diagnostics16121755/s1, Table S1: Pairwise binary classification AUC for all 15 non-empty plasma biomarker subsets; Table S2: Incremental contribution of clinical scales to three-class AUC-OVR; Table S3: Three-class multiclass Brier scores; Table S4: Pairwise binary Brier scores; Table S5: Aggregated confusion matrices for the best-performing model in each feature set; Table S6: Comparison of included and excluded ADNI participants; Table S7: Missing-data sensitivity analysis results; Figure S1: SHAP feature importance for the Random Forest fusion model; Figure S2: One-vs.-rest calibration curves; Supplementary Note S1: Discrimination vs. Calibration [17]; Supplementary Note S2: CN vs. MCI Classification Difficulty; Supplementary Note S3: Missing-data sensitivity analysis.

## Abbreviations

The following abbreviations are used in this manuscript:

AD	Alzheimer’s Disease
ADNI	Alzheimer’s Disease Neuroimaging Initiative
APOE4	Apolipoprotein E ε4 allele
AUC	Area Under the Receiver Operating Characteristic Curve
AUC-OVR	AUC, One-vs.-Rest (multiclass)
Aβ42/40	Amyloid-β 42/40 Ratio
CDR-SB	Clinical Dementia Rating Sum of Boxes
CN	Cognitively Normal
CNTN	Center for Neurodegeneration and Translational Neuroscience
CSF	Cerebrospinal Fluid
CV	Cross-Validation
FAQ	Functional Activities Questionnaire
GFAP	Glial Fibrillary Acidic Protein
LR	Logistic Regression
MCI	Mild Cognitive Impairment
ML	Machine Learning
MMSE	Mini-Mental State Examination
NfL	Neurofilament Light Chain
PET	Positron Emission Tomography
pT217	Plasma Phosphorylated Tau-217
RF	Random Forest
ROC	Receiver Operating Characteristic
SHAP	SHapley Additive exPlanations
SVM	Support Vector Machine
XGBoost	eXtreme Gradient Boosting
