# Supplementary material for: Machine Learning-Based Multiclass Classification of Cognitive Stages Using Plasma Biomarkers, Clinical Assessments, and Genetic Features: A Repeated, Nested Cross-Validation Study in ADNI with External Evaluation in CNTN
Source: Diagnostics (Basel). 2026 Jun 6;16(12):1755. doi: 10.3390/diagnostics16121755 (PMC13298147; doi:10.3390/diagnostics16121755)
Supplement: Supplementary file 1 [file diagnostics-16-01755-s001.zip › diagnostics-4332323-supplementary.pdf]

Supplementary Materials: Machine Learning-Based Multiclass Classification of Cognitive Stages Using Plasma Biomarkers, Clinical Assessments, and Genetic Features: A Repeated, Nested Cross-Validation Study in ADNI with External Evaluation in CNTN

Jiayuan Xu [1] and Fumie Costen [1,*]

1. Supplementary Tables

Table S1. Pairwise binary classification AUC for all 15 non-empty plasma biomarker subsets, evaluated with fixed background features (age, sex, education, APOE4 allele count; Random Forest; repeated nested cross-validation, 15 outer folds).

Biomarker Subset	AUC-OVR (95% CI)	CN vs. MCI AUC (95% CI)	CN vs. AD AUC (95% CI)	MCI vs. AD AUC (95% CI)
Single biomarker				
pT217 only	0.7392 ± 0.0165	0.6668 ± 0.0287	0.9084 ± 0.0127	0.7197 ± 0.0229
Aβ42/40 only	0.6237 ± 0.0168	0.5921 ± 0.0343	0.6877 ± 0.0210	0.5801 ± 0.0312
NfL only	0.6926 ± 0.0130	0.6111 ± 0.0223	0.8240 ± 0.0133	0.6714 ± 0.0198
GFAP only	0.6339 ± 0.0135	0.5457 ± 0.0247	0.7616 ± 0.0236	0.5838 ± 0.0298
Two-biomarker panels				
pT217 + NfL	0.7531 ± 0.0155	0.6875 ± 0.0258	0.9238 ± 0.0112	0.7665 ± 0.0188
pT217 + GFAP	0.7322 ± 0.0167	0.6611 ± 0.0262	0.9022 ± 0.0139	0.7283 ± 0.0208
pT217 + Aβ42/40	0.7374 ± 0.0153	0.6841 ± 0.0301	0.9045 ± 0.0139	0.7221 ± 0.0224
NfL + GFAP	0.6767 ± 0.0125	0.5781 ± 0.0142	0.8317 ± 0.0155	0.6650 ± 0.0257
Aβ42/40 + NfL	0.6924 ± 0.0134	0.6412 ± 0.0249	0.8287 ± 0.0149	0.6739 ± 0.0225
Aβ42/40 + GFAP	0.6463 ± 0.0148	0.5805 ± 0.0257	0.7659 ± 0.0215	0.6111 ± 0.0303
Three-biomarker panels				
pT217 + NfL + GFAP	0.7468 ± 0.0157	0.6800 ± 0.0220	0.9183 ± 0.0114	0.7689 ± 0.0175
pT217 + Aβ42/40 + NfL	0.7482 ± 0.0132	0.7054 ± 0.0222	0.9193 ± 0.0117	0.7645 ± 0.0172
pT217 + Aβ42/40 + GFAP	0.7315 ± 0.0163	0.6784 ± 0.0322	0.8993 ± 0.0141	0.7310 ± 0.0206
Aβ42/40 + NfL + GFAP	0.6807 ± 0.0133	0.6197 ± 0.0220	0.8324 ± 0.0139	0.6725 ± 0.0207
Full panel (equivalent to biomarker-plus-demographic-genetic set)				
pT217 + Aβ42/40 + NfL + GFAP	0.7455 ± 0.0150	0.6972 ± 0.0261	0.9153 ± 0.0134	0.7588 ± 0.0147

Results are mean AUC $\pm$ 95% CI ($= 1.96 \times SD/\sqrt{15}$) across 15 outer folds. AUC-OVR = macro-averaged one-vs-rest AUC for three-class classification. Background features included in all models. Abbreviations: CN, cognitively normal; MCI, mild cognitive impairment; AD, Alzheimer's disease; AUC, area under the ROC curve; CI, confidence interval.

Table S2. Incremental contribution of individual and combined clinical assessment scales to three-class (CN/MCI/AD) AUC-OVR, evaluated by sequentially adding MMSE, CDR-SB, and FAQ to the biomarker-plus-demographic-genetic feature set (15 outer folds).

**Feature Set**	***n* Features**	**AUC-OVR**	**95% CI (±)**
Baseline			
Biomarker + Demo + Genetic (baseline)	8	0.7455	0.0150
Single scale added			
+ MMSE	9	0.8421	0.0098
+ CDR-SB	9	0.9502	0.0065
+ FAQ	9	0.8904	0.0073
Two scales added			
+ MMSE + CDR-SB	10	0.9515	0.0067
+ MMSE + FAQ	10	0.9051	0.0063
+ CDR-SB + FAQ	10	0.9524	0.0062
All three scales added			
+ All 3 scales (Fusion)	11	0.9538	0.0056

Results are mean AUC-OVR ± 95% CI across 15 outer folds. CDR-SB alone accounts for the largest single-scale AUC increment (+0.2047), followed by FAQ (+0.1449) and MMSE (+0.0966).

Table S3. Three-class (CN/MCI/AD) multiclass Brier scores by feature set and classifier (repeated nested cross-validation, 15 outer folds). Lower values indicate better calibration. A no-skill classifier scores ≈ 0.222 for a balanced three-class problem.

**Feature Set**	**Classifier**	**Brier Score**	**95% CI (±)**
Clinical-only (3 features)			
	SVM	0.0659	0.0040
	Logistic Regression	0.0675	0.0036
	Random Forest	0.0698	0.0041
	XGBoost	0.0709	0.0042
Fusion (11 features)			
	SVM	0.0706	0.0042
	XGBoost	0.0699	0.0035
	Random Forest	0.0728	0.0034
	Logistic Regression	0.0791	0.0035
Biomarker + Demographic + Genetic (8 features)			
	SVM	0.1690	0.0053
	Random Forest	0.1706	0.0043
	Logistic Regression	0.1764	0.0054
	XGBoost	0.1731	0.0048

Results are mean ± 95% CI across 15 outer folds, sorted by ascending mean Brier score within each feature set.

Table S4. Pairwise binary Brier scores for the fusion feature set across three diagnostic contrasts (15 outer folds). A no-skill classifier scores ≈ 0.25 for a balanced binary problem.

Task	Classifier	Brier Score	95% CI (±)
CN vs. AD			
	XGBoost	0.0051	0.0023
	Random Forest	0.0059	0.0017
	SVM	0.0087	0.0030
	Logistic Regression	0.0133	0.0041
MCI vs. AD			
	Random Forest	0.0699	0.0082
	SVM	0.0708	0.0080
	Logistic Regression	0.0708	0.0078
	XGBoost	0.0766	0.0103
CN vs. MCI			
	SVM	0.0966	0.0121
	Logistic Regression	0.1001	0.0079
	Random Forest	0.0984	0.0071
	XGBoost	0.0962	0.0080

Results are mean ± 95% CI across 15 outer folds. CN vs. AD achieves near-perfect calibration (≤ 0.0102). CN vs. MCI has the highest Brier scores (≈ 0.09–0.10), reflecting the difficulty of the early-stage boundary.

Table S5. Aggregated confusion matrices (summed across 15 outer folds) for the best-performing model configurations in each feature set: fusion/Random Forest, clinical-only/Logistic Regression, and biomarker+demographic+genetic/Random Forest. Each participant appears in exactly 3 test folds.

Model	True	Pred CN	Pred MCI	Pred AD	Recall	Precision
Fusion / RF						
	CN	784	104	0	88.3%	90.6%
	MCI	81	368	55	73.0%	70.6%
	AD	0	49	524	91.4%	90.5%
Clinical / LR						
	CN	821	67	0	92.5%	88.7%
	MCI	102	359	43	71.2%	76.1%
	AD	3	46	524	91.4%	92.4%
Bio+Demo+Gen / RF						
	CN	715	99	74	80.5%	68.1%
	MCI	244	100	160	19.8%	38.3%
	AD	91	62	420	73.3%	64.2%

Counts are aggregated across all 15 outer folds (5-fold × 3 repeats); each participant appears in exactly 3 test folds. Recall = TP/(TP+FN) per row; precision values are shown for the diagonal class of each column. The fusion model's main weakness is MCI recall (73.0%), reflecting the clinical difficulty of distinguishing early cognitive decline from normal aging.

Table S6. Comparison of included ($n = 655$) versus excluded ($n = 92$) ADNI participants. Participants were excluded solely because of missing (sentinel-coded) plasma biomarker values; plasma biomarkers are therefore unavailable for the excluded group and are not compared, whereas all other variables were available for both groups.

Variable	Included ($n = 655$)	Excluded ($n = 92$)	SMD	p-value
Age (years)	77.9 ± 7.9	75.5 ± 7.9	−0.31	0.007
Education (years)	16.3 ± 2.5	16.2 ± 2.5	−0.08	0.487
MMSE score	25.6 ± 5.6	27.1 ± 4.0	+0.30	0.002
CDR-SB	2.5 ± 3.7	1.5 ± 2.7	−0.31	0.002
FAQ total	6.4 ± 9.2	3.8 ± 7.1	−0.31	0.002
Female sex, n (%)	296 (45.2%)	56 (60.9%)	+0.32	0.007
APOE4 carrier, n (%)	299 (45.6%)	29 (31.5%)	−0.29	0.015

Continuous variables: mean ± SD, independent-samples (Welch) t-test. Categorical variables: n (%), Pearson chi-square test. SMD, standardized mean difference (Cohen's d for continuous variables, Cohen's h for proportions); $|\text{SMD}| > 0.2$ indicates a small effect. Plasma biomarkers (pT217, Aβ42/40, NfL, GFAP) are omitted because their absence (ADNI sentinel codes) defines the excluded group. Excluded participants were younger, more often female, less likely to carry APOE4, and less cognitively impaired (higher MMSE, lower CDR-SB and FAQ), indicating modest selection toward a more clinically impaired analytic sample. All effect sizes were small ($|\text{SMD}| \leq 0.32$).

2. Supplementary Notes

Supplementary Note S1: Discrimination vs. Calibration

AUC measures a classifier's ability to rank individuals (discrimination), whereas Brier scores additionally capture the agreement between predicted probabilities and observed outcomes (calibration). A model can achieve high AUC while being poorly calibrated if its probability estimates are systematically over- or under-confident. Tables S3 and S4 present Brier scores alongside AUC to address this distinction. For the fusion feature set, XGBoost achieves both the highest AUC-OVR (0.9559) and the lowest multiclass Brier score (0.0699), indicating that its probability estimates are well-calibrated in addition to being discriminative. The clinical-only set shows a similar pattern, with SVM yielding the best Brier score (0.0659). The biomarker-only set has substantially higher Brier scores (best: 0.1690 for SVM), consistent with its lower AUC and reflecting both poorer discrimination and less reliable probability estimates for this feature set. In pairwise comparisons, CN vs. AD shows near-perfect calibration (Brier ≈ 0.005–0.006 for XGBoost and RF), while CN vs. MCI has the highest Brier scores (≈ 0.10), confirming that MCI classification remains the most difficult task not only in discrimination but also in producing confident probability assignments. Reporting calibration alongside discrimination follows established best practice for predictive models [22].

Supplementary Note S2: CN vs. MCI Classification Difficulty

Distinguishing cognitively normal (CN) individuals from those with mild cognitive impairment (MCI) is the most clinically relevant yet most challenging classification task, as evidenced by consistently lower AUCs and higher Brier scores across all feature sets. Even the clinical-only model, which includes MMSE, CDR-SB, and FAQ, does not fully resolve CN vs. MCI, with MCI recall of only 71.2% (Table S5). This reflects the inherent overlap in clinical presentation between normal aging and early cognitive decline, particularly in individuals with high cognitive reserve. Plasma biomarkers alone achieve an MCI recall of only 19.8%, with the majority of MCI cases (244/504 test appearances) misclassified as CN. The fusion model improves MCI recall to 73.0% by combining biomarker and clinical information, but a substantial proportion of MCI cases remain misclassified as either CN (81/504) or AD (55/504).

Supplementary Note S3: Missing-Data Sensitivity Analysis

The main analysis excluded 92 participants whose plasma biomarkers were ADNI sentinel-coded (missing; see Supplementary Table S6). To assess whether this exclusion biased the three-class results, we performed a missing-data sensitivity analysis. For each excluded participant, the missing plasma biomarker values (pT217, Aβ42/40, NfL, GFAP) were imputed with the median of the corresponding biomarker in the analytic training sample ($n = 655$); all available demographic, genetic, and clinical values were retained unchanged. The 92 imputed participants were then added to the analytic sample (augmented $n = 747$; CN = 347, MCI = 194, AD = 206), and the three-class biomarker-plus-demographic-genetic model was re-evaluated under the identical repeated nested cross-validation protocol (5-fold $\times$ 3 repeats = 15 outer folds).

Table S7. Missing-data sensitivity analysis: three-class (CN/MCI/AD) AUC-OVR for the biomarker-plus-demographic-genetic model under the main analytic sample ($n = 655$) versus the augmented sample with median-imputed excluded participants ($n = 747$); repeated nested cross-validation, 15 outer folds.

**Classifier**	**Main analysis ($n = 655$) AUC-OVR (95% CI)**	**Augmented, imputed ($n = 747$) AUC-OVR (95% CI)**
Logistic Regression	0.7384 ± 0.0200	0.7455 ± 0.0125
Random Forest	0.7455 ± 0.0150	0.7429 ± 0.0136
SVM	0.7356 ± 0.0197	0.7441 ± 0.0121
XGBoost	0.7388 ± 0.0157	0.7426 ± 0.0120

Results are mean AUC-OVR ± 95% CI across 15 outer folds. After imputation the AUC-OVR changed by at most 0.009 across classifiers (primary Random Forest model: 0.7455 → 0.7429), well within the overlapping 95% confidence intervals.

Across all four classifiers (Table S7), the three-class AUC-OVR after imputation was within 0.009 of the corresponding main-analysis value and well inside the overlapping 95% confidence intervals (primary Random Forest model: 0.7455 → 0.7429, a change of −0.003). The central finding, a plasma-based three-class performance level near 0.74, is therefore robust to the exclusion of participants with missing biomarker data, and median imputation of the excluded cases does not materially alter the results.

3. Supplementary Figures

(A)

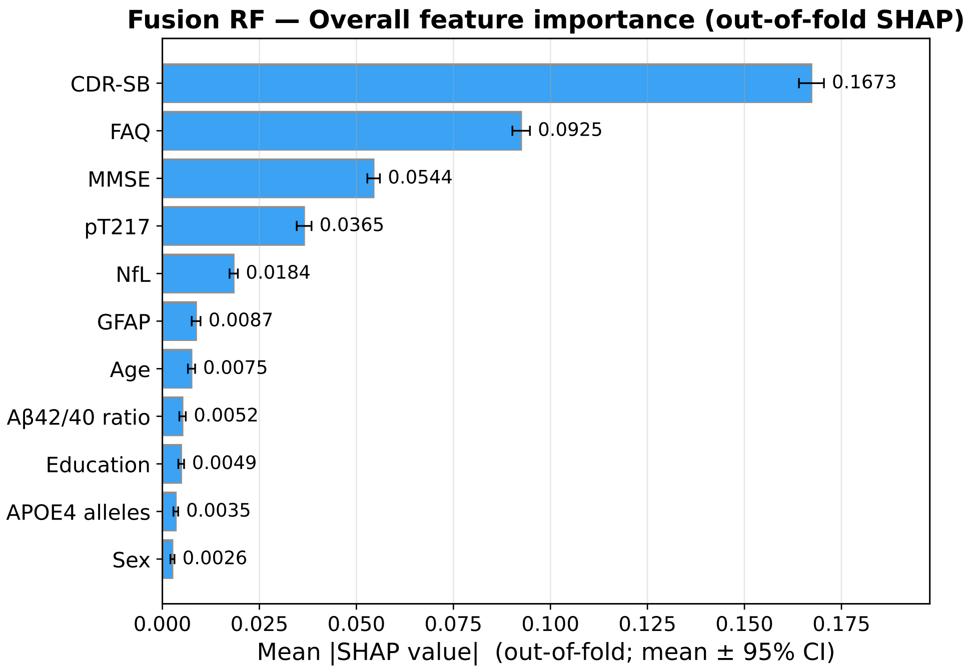

(B)

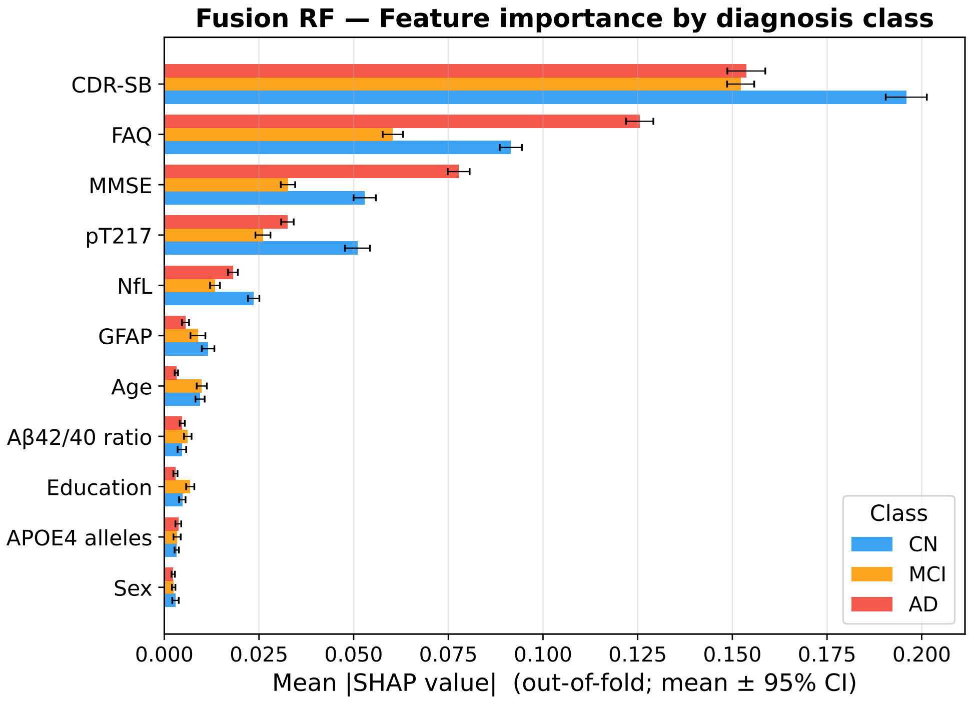

Figure S1. SHAP feature importance for the fusion Random Forest model (11 features), aggregated across the 15 outer cross-validation folds (mean ± 95% CI; error bars). (**A**) Overall mean absolute SHAP value averaged across CN, MCI, and AD classes. CDR-SB accounts for 41.7% of total importance, followed by FAQ (23.0%) and MMSE (13.6%); the three clinical scales combined contribute 78.3%. pT217 is the highest-ranked plasma biomarker at 9.1%. (**B**) Per-class SHAP importance showing that CDR-SB dominates across all three diagnostic classes.

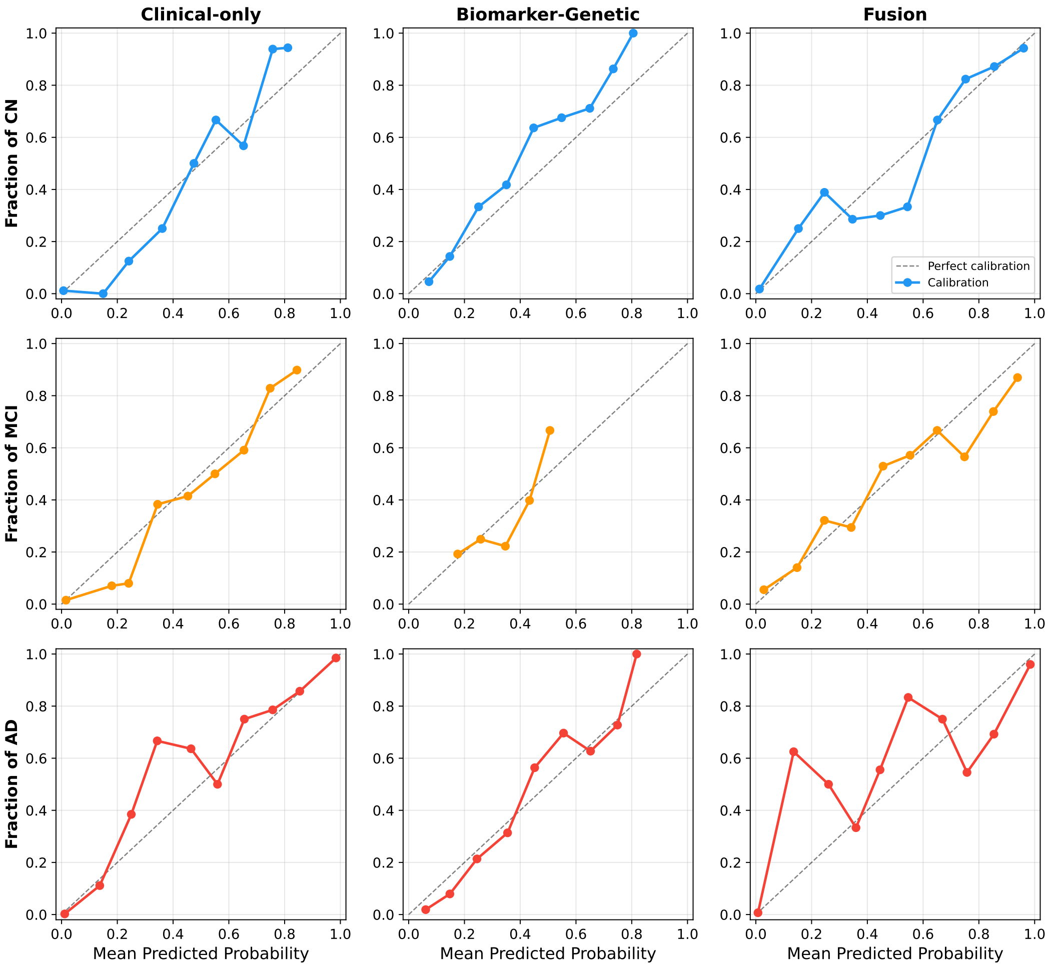

Figure S2. One-vs-rest calibration curves for the best-performing model in each feature set: Clinical-Only (Logistic Regression), Biomarker+Demographic+Genetic (Random Forest), and Fusion (Random Forest). Predicted probabilities were aggregated across all 15 outer cross-validation folds and binned into 10 equal-width intervals. The dashed diagonal represents perfect calibration. The clinical-only and fusion models show good calibration for CN and AD classes. The biomarker-only model shows moderate miscalibration for MCI, consistent with the limited separability of this intermediate class using plasma biomarkers alone.

References

22. Van Calster, B.; McLernon, D.J.; van Smeden, M.; Wynants, L.; Steyerberg, E.W. Calibration: The Achilles heel of predictive analytics. *BMC Med.* **2019**, *17*, 230. https://doi.org/10.1186/s12916-019-1466-7.
